# Expanding the Toolbox: Mangrove Use by Spotted Eagle Rays (*Aetobatus ocellatus*) in the Maldives Revealed Through Drone‐Based Observation

**DOI:** 10.1002/ece3.73832

**Published:** 2026-06-11

**Authors:** Giulia Senna, Jacopo Gobbato, Yohan Didier Louis, Giorgia Gobbato, Federico Cerri, Jana Pažin, Andrea Parmegiani, Rossella Nicolai, Shazla Mohamed, Paolo Galli

**Affiliations:** ^1^ Department of Earth and Environmental Sciences (DISAT) University of Milano‐Bicocca Milan Italy; ^2^ MaRHE Center (Marine Research and High Education Center) Magoodhoo Island Republic of Maldives; ^3^ NBFC (National Biodiversity Future Center) Palermo Italy; ^4^ Mubadala Arabian Center for Climate and Environmental Sciences (Mubadala ACCESS) New York University Abu Dhabi Abu Dhabi UAE; ^5^ The Maldives National University Malé Maldives

**Keywords:** batoids, citizen science, elasmobranch, Indo‐Pacific Ocean, nursery, UAV

## Abstract

Mangrove ecosystems are critical coastal habitats that serve as nurseries for a wide range of marine species; yet their role in elasmobranchs' life cycle and conservation remains poorly documented, particularly in regions where mangroves are limited and threatened. Here, we report the first scientific record of the Spotted Eagle Ray *Aetobatus ocellatus* (Kuhl, 1823) within a mangrove‐surrounded embayment of Baarah Island, Republic of the Maldives. Observations were made using a commercially available unmanned aerial vehicle (UAV), a technology increasingly adopted within citizen science initiatives, which enabled the observation of four individuals and the recording of one juvenile. Recordings were taken during two consecutive manual overflights (~50 min) over a 1.5 km^2^ area, during high tide and with sunny‐to‐slightly overcast conditions. This finding supports the hypothesis that mangrove habitats may serve as refuge or nursery areas for elasmobranch early life stages, as well as for *A. ocellatus* , despite being rarely described as habitats for eagle rays. Our findings suggest that Maldivian mangroves, though spatially limited and highly threatened, may hold greater ecological importance for batoids than previously acknowledged, thereby supporting current environmental conservation plans implemented in Baarah Island while simultaneously serving as a baseline for further safeguarding actions. This study further highlights how accessible aerial technologies can enhance the detection of elusive species in challenging environments and help fill critical knowledge gaps relevant to elasmobranch conservation.

## Graphical abstract

This study reports the first scientific record of the spotted eagle ray *Aetobatus ocellatus* within a mangrove embayment in the Republic of the Maldives, documented through drone‐based observations. We observed four individuals, including one juvenile, that we recorded using an unmanned aerial vehicle (UAV), highlighting mangrove habitats as potential refuges or nurseries for the early life stages of this species.

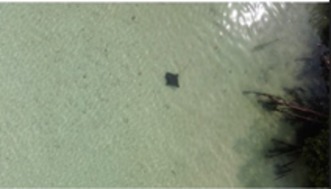

## 1 Introduction

Mangrove ecosystems are among the most productive coastal habitats worldwide and play a fundamental ecological role in tropical and subtropical regions (Alongi 2002; Donato et al. 2011), providing key ecosystem services such as shoreline stabilisation, carbon sequestration, and nutrient cycling (Alongi 2014; Cerri et al. 2024; Costanza et al. 1997). Importantly, mangroves serve as critical nursery habitats for juvenile fishes and invertebrates, offering shelter from predators, high food availability, and environmental conditions that enhance early survival and growth (Nagelkerken et al. 2008; Sheaves et al. 2015). Among the taxa benefiting from mangrove‐associated nurseries, elasmobranchs have received increasing attention, as juvenile sharks and rays frequently use shallow, protected environments such as mangrove lagoons and creeks, where reduced predation risk and abundant benthic prey support early life stages (Heupel et al. 2007; Martins et al. 2020). Despite this recognised importance, mangrove forests are affected by a number of threats including pollution (Cerri et al. 2025; De et al. 2023), anthropogenic pressure and climate‐driven stressors (GMA 2024), that have led to the rapid global decline of these ecosystems (Estoque et al. 2018; Giri et al. 2008; Goldberg et al. 2020), while the ecological role of these habitats for many elasmobranch species remains poorly documented, largely because turbidity, shallow depths, and the cryptic behaviour of juveniles hinder direct observation, particularly in remote or logistically complex regions (Duke et al. 2007; Friess et al. 2019).

In the Maldives, mangrove habitats are extremely limited in extent, fragmented, and classified as highly threatened, covering a total area estimated at less than 700 ha across the entire archipelago (Cerri et al. 2024). Unlike continental mangroves, Maldivian systems are not connected to riverine inputs and can be classified as open or closed ecosystems depending on their degree of tidal connection to the ocean (Cerri et al. 2024). Baarah Island, located in Haa Alifu Atoll in the northern Maldives, hosts one of the few relatively intact semi‐enclosed mangrove embayments in the archipelago, characterised by a shallow, sheltered habitat with limited water exchange and persistent mangrove fringing, broadly associated with elasmobranch nursery use in other Indo‐Pacific regions (Heupel et al. 2007; Martins et al. 2020). These areas remain partially or fully flooded throughout the tidal cycle, and while depth can increase in some sections, the overall water level rarely allows comfortable cruising by large predators, providing a safeguarded habitat for elasmobranch juveniles (Heupel and Hueter 2002). Moreover, although tourist interest in mangrove environments is growing, access to muddy and turbid mangrove embayments remains discouraged, as the presence of stonefish and stingrays can pose a health concern (Warrell 2013), thereby limiting in‐water human disturbance. Despite their potential ecological importance, especially for juvenile marine fauna, Maldivian mangroves remain poorly studied, and their role for elasmobranch species is largely unknown.

Recent technological advances have begun to overcome these observational limitations. A growing array of instruments is now available to the general public, enabling meaningful scientific contributions. In environmental science, low‐cost sensors and tailored kits are now available to monitor air pollutants, weather, collect microplastics, and more (Camprodon et al. 2019; Mahajan et al. 2020; Paradinas et al. 2021; Ripoll et al. 2019). The development of open digital platforms has facilitated the collection and processing of data derived from citizen science initiatives, and it has ensured that such data is returned, readily available, to the public, as Zooniverse for astronomical data (Cox et al. 2015) or iNaturalist for ecological and natural sciences (Nugent 2018). Furthermore, technological tools once considered inaccessible beyond specialist research, have now entered mainstream use, including camera traps (Green et al. 2020), mobile apps (Vohland et al. 2021), 3D printers (Horvath and Cameron 2015), and even drones. These initiatives, encompassed in the practice of citizen science, are attracting significant attention and growing recognition within the field of ecology due to the substantial volume of data they provide to researchers. These initiatives facilitate the exploration of various species following appropriate control and validation measures, while also promoting their application on a global scale across several challenging domains of marine research, such as the distribution or behaviour of elusive and rare species (Araujo et al. 2020; Bargnesi et al. 2020; Gobbato et al. 2024; Parmegiani et al. 2023; Séguigne et al. 2023; Siena et al. 2025; Whitehead et al. 2025).

In particular, unmanned aerial vehicles (UAVs), commonly referred to as drones, have received particular attention in recent years for wildlife monitoring across several taxa, including mammals (Berezina et al. 2024), birds (Brisson‐Curadeau et al. 2025), and fish (Raoult et al. 2018), in both land and marine environments. Due to the possibility to acquire systematic and continuous data with high spatial and temporal distribution (Linchant et al. 2015), UAVs have proven to be particularly useful for the assessment of hard‐to‐locate and elusive species such as elasmobranchs and increase the amount of data that is available on their behaviours, populations, and occurrences (Dirzo et al. 2014). In the context of shallow mangrove habitats, UAV‐based monitoring is not only a convenient alternative to in‐water surveys but also the most practical non‐invasive approach available in response to the combination of turbid water, dense mangrove fringing, and shallow depths that preclude effective diver‐based observation. The increasing availability of consumer‐grade drones, as evidenced by the growing revenue of drone manufacturers (www.statista.com) and the expanding reach of citizen science initiatives, could represent a valuable resource for habitat and species monitoring. Nevertheless, the application of UAV‐based observations in mangrove systems remains geographically uneven, and their use for detecting elasmobranchs in enclosed mangrove embayments in the Maldives is almost absent.

Numerous batoid (rays and skates) species have already been studied through the contribution of citizen science (Germanov and Marshall 2014; Giareta et al. 2025; Glaus et al. 2024), including the common but elusive Spotted Eagle Ray (*Aetobatus ocellatus*, Kuhl 1823) (Araujo et al. 2024). *Aetobatus ocellatus* is one of five species in the family Aetobatidae (Agassiz, 1858) (White and Naylor 2016), distributed throughout the whole Indo‐West Pacific region and distinguished by its dark green‐grey dorsal colouration with a variable pattern of white spots. Its disc width (DW) is the largest in the family, reaching up to 300 cm, and it inhabits coastal environments, where it primarily feeds on bottom‐dwelling molluscs and crustaceans (Last et al. 2016). The species is ovoviviparous, with litters generally comprising up to four pups. DW at birth ranges from 18 to 26 cm, and sexual maturity is considered reached at DW of 110–160 cm (Last et al. 2010). Due to its slow growth and late maturity, *A. ocellatus* populations have undergone severe declines over the last few decades, resulting in listing as Endangered on the IUCN Red List of Threatened Species (Finucci et al. 2023). As their numbers decline, it is essential to identify critical habitats for this species to implement effective conservation strategies, but their inherently elusive behaviour poses challenges to obtaining comprehensive ecological data. The rise in availability of technological tools, such as UAVs, for the general public represents a considerable advantage for the scientific community, which can exploit the images produced to infer scientific data. Consequently, contributions from citizen science are to enhance data collection efforts and fill knowledge gaps in the ecology of this species, particularly in remote locations where the easiest and most frequent access is granted to tourists.

This study reports the first scientific record of *A. ocellatus* in one of the semi‐enclosed, sheltered mangrove embayments of the Maldives. The record was obtained using a commercially available drone supported by citizen science contributions. The footage was used to describe the morphological features for species identification, provide measurement estimates for juvenile classification, discuss the environmental context of the embayment and its potential function as a nursery or refuge habitat, and evaluate the methodological strengths and limitations of UAV‐based elasmobranch monitoring in fragmented coastal habitats. These findings represent novel baseline data on batoid behaviour and habitat use, endorse the use of publicly available technologies for scientific records, and provide novel evidence supporting the role of Maldivian mangroves as potential nursery habitats for juvenile eagle rays, contributing to the conservation relevance of these rare and threatened ecosystems.

## 2 Material and Methods

**FIGURE 1:**
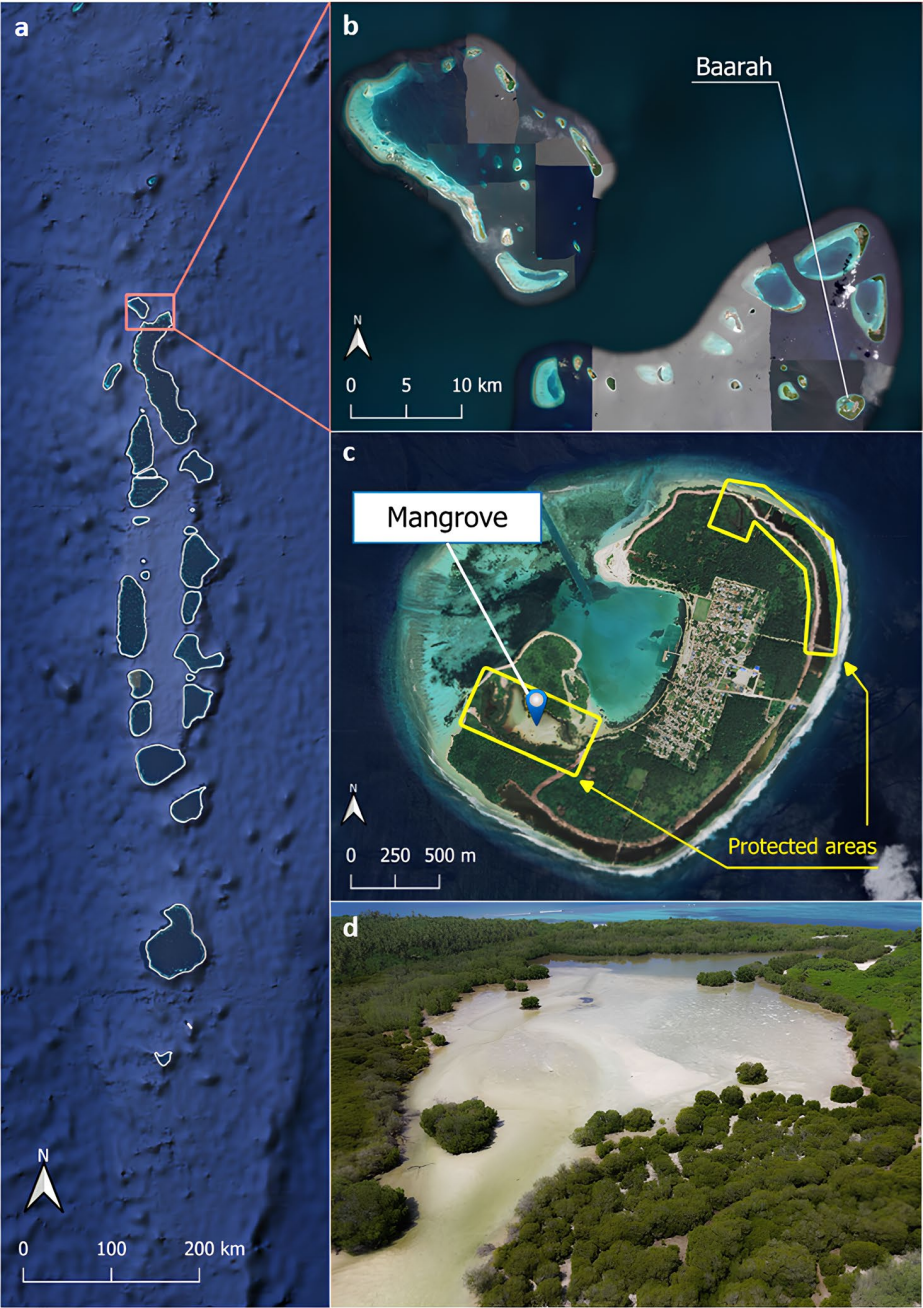
Geographical contextualization of the study area. (a) Localisation of Haa Alifu atoll within the Republic of the Maldives; (b) detailed view of the atoll's extension with relative position of Baarah island; (c) detail of Baarah island with location of Environmental Protection Agency areas (EPA, yellow outlines) and the mangrove site of the study (6°48′52″N, 73°12′08″E); (d) Flight view of the study area.

Video footage was recorded in a mangrove embayment at Baarah, Haa Alifu Atoll, Republic of the Maldives (6°48′52″N, 73°12′08″E; Figure 1) in November 2024. The embayment is characterised by a diurnal tidal regime typical of the Maldivian archipelago, with a tidal range of approximately 0.8–1.0 m. The area is subject to anthropogenic pressures, including proximity to a local settlement and coastal infrastructure, and recreational boat traffic associated with local tourism; however, in‐water human access to the embayment is generally limited due to the muddy substrate and the presence of potentially hazardous benthic fauna (Warrell 2013). The total surface area of the embayment was calculated in Google Earth Pro using the mangrove tree line as a boundary, yielding approximately 1.5 km^2^.

The video records were taken with a DJI Mini 3 quadcopter (DJI, China) during two consecutive flights of approximately 25 min each. Flights were carried out in the late morning to cover the peak of high tide and the beginning of the ebb phase. During deployment, weather conditions changed from strong sunshine, producing glint on the water surface, to slightly overcast conditions, with wind speed consistently below 7 km.

The camera was set to F/1.7, ISO 100, and Digital Zoom 1.0, and video was recorded in 4 K HDR. Visibility at the site was considered optimal as, given the limited water depth (estimated below 1.5 m) and lack of strong currents, the majority of benthic features were clearly visible in the recorded videos. Individuals were located and recorded during manual overflight with the camera pointed vertically downward to minimise distortion and maximise measurement accuracy. Additional video materials, including close‐up images and details of the encounter, were recorded by citizen scientists from the mangrove tree line and adjacent shoreline using a GoPro Hero12 action camera (GoPro, USA) and smartphone cameras.

For size estimation, a 5 m measuring tape was fixed along the mangrove tree line at the water surface as a spatial reference for DW and total length (TL) measurement. The flight altitude at the time of measurement was 10 m above ground level (AGL), selected to minimise camera distortion and maintain adequate spatial resolution (Whitehead et al. 2022). To account for measurement error, five different frames were used to calculate DW and TL, and the mean of the measurements was noted down. There are intrinsically two potential sources of error in UAV‐based size estimation in shallow aquatic environments: the difference in focal plane between the reference tape, positioned at the water surface, and the ray, located at depth; and the optical refraction at the air‐water interface, which causes submerged objects to appear shallower and proportionally larger than their true size when viewed from directly above. Cruising depth of the eagle ray specimen was considered negligible due to shallow water (< 0.5 m) when in proximity of mangrove roots based on visual assessment of the footage and the shallow nature of the site; nonetheless, a positive size distortion of 33%–34% caused by seawater refraction was accounted for according to Shortis (2015). Final size estimates are reported with an associated uncertainty range reflecting the combined sources of measurement error.

All video files were uploaded and imported into the open‐source software BORIS (Behavioural Observation Research Interactive Software; Friard and Gamba 2016) to extract individual frames and to perform morphometric measurements (DW and TL). For this study, individuals were classified as juveniles based on size‐at‐maturity thresholds reported in the literature. Specifically, *A. ocellatus* was considered juvenile when DW was below half sexual maturity size, corresponding to < 150 cm DW for males and < 110 cm DW for females (Bassos‐Hull et al. 2014; Finucci et al. 2023; White et al. 2010).

## 3 Results

**FIGURE 2:**
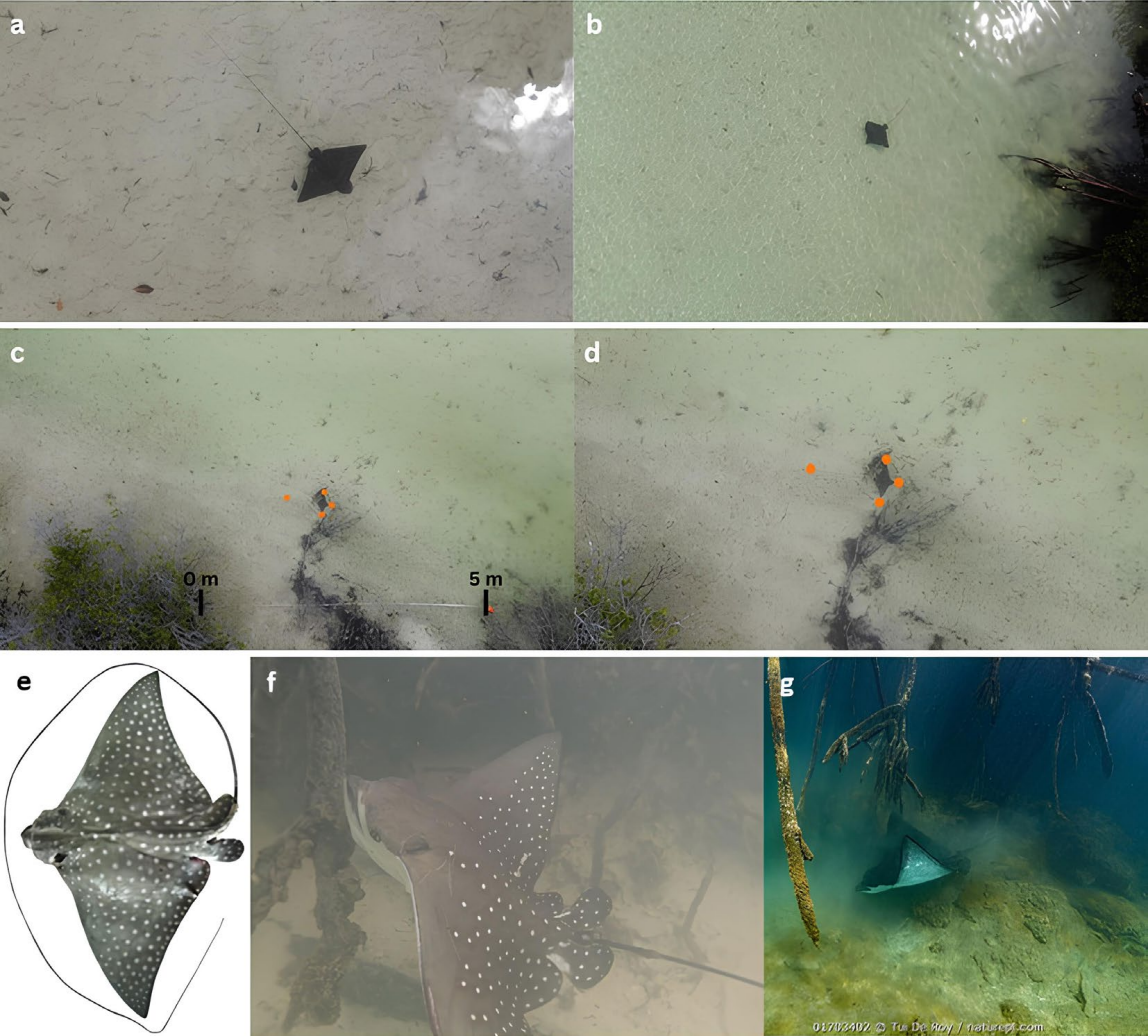
Images documenting *Aetobatus ocellatus* in the mangrove habitats. (a) Close‐up view of the specimen *A. ocellatus* for correct species identification (height from ground: 2.4 m); (b) Individual of *A. ocellatus* cruising in proximity of mangrove roots; (c) Length measurements of the specimen relative to the adjacent tape. (d) Details of the points used for DW and TL measurements; (e) Illustration of *A. ocellatus* showing key morphological features (adapted from Last et al. 2010). (f) Interaction of an *A. ocellatus* individual with mangrove roots; (g) “Spotted eagle ray ( *Aetobatus narinari* ) resting in mangrove inlet, Venecia, Santa Cruz Island, Galapagos Islands, Ecuador. Pacific Ocean.” (photo credit: Tui De Roy) from Nature Picture Library.

A total of four *A. ocellatus* individuals, approximately the same size, were observed within the mangrove embayment of Baarah, Haa Alifu atoll (Figure 1), but only two were captured together in video (Supporting Information S1). Species identification was based on the following morphological features clearly visible in the UAV and GoPro footage: a rounded cephalic fin margin, a dark dorsal disc with a clearly visible pattern of white spots, a broadly rhomboid disc shape, and an elongated whip‐like tail substantially exceeding DW (Last et al. 2016; White et al. 2010; Figure 2). The only morphologically similar and potentially confounding species, *Aetobatus narinari* (Euphrasen, 1790), is limited to the Atlantic Ocean (Richards et al. 2009), further supporting the identification.

Among the four individuals observed, three quickly moved out of the mangrove area into the open ocean upon detecting in‐water human presence, while one individual remained stationary inside the embayment. The footage presented here reports a small eagle ray with an estimated DW of 19.7–20 ± 1.0 cm and TL of 40.2–40.8 ± 1.5 cm, measured from the snout to the tip of the tail, after correction for seawater refraction following Shortis (2015); both measurements carry an associated uncertainty reflecting combined sources of measurement error. The individual showed light scarring on the dorsal side (Figure 2). Based on the measured sizes, the individual was classified as a juvenile, but the sex could not be unequivocally determined from the available video footage, despite the presence of lateral views and close‐up imagery (Figure 2f; Supporting Information S2).

## 4 Discussion

This study represents the first scientific record of *A. ocellatus* within a mangrove system in the Republic of the Maldives, documenting the presence of a juvenile individual in a shallow enclosed habitat surrounded by mangrove vegetation. The habitat of pelagic eagle rays (Aetobatidae) and eagle rays (Myliobatidae; Bonaparte, 1838) extends from marine waters to channels, lagoons, and even estuaries, on flat bottoms of sand or rock (DeGroot et al. 2020). Although the presence of (pelagic) eagle rays in mangroves or their proximity has been suggested before (Cerutti‐Pereyra et al. 2018; Flowers et al. 2017), scientific literature remains scarce, despite the well‐established role of shallow and sheltered coastal habitats as nurseries for juveniles of several fish (Nagelkerken et al. 2008; Whitfield 2017) and elasmobranch species (Heupel et al. 2019; White and Potter 2004). Indeed, while a non‐scientific record of pelagic eagle rays cruising close to mangrove roots is available online (Figure 2g), to the best of our knowledge, no previous scientific report has documented these animals making use of semi‐protected and enclosed mangrove bays. The environmental characteristics of the Baarah embayment, including shallow depth, semi‐enclosed morphology, limited water exchange, mangrove fringing, and proximity to open reef habitat, are broadly consistent with the habitat features linked to elasmobranch nursery use (Heupel et al. 2007; Martins et al. 2020). Reduced predation pressure from large pelagic predators, structural shelter provided by mangrove root architecture, and the availability of benthic prey are the primary characteristics proposed to favour juvenile elasmobranch residency in such environments (Heupel et al. 2007; Martins et al. 2020). The observation provides the first empirical basis for directing systematic monitoring efforts toward this and similar sites in the Maldives.

In the Maldives, mangrove areas can be classified as closed, when constituted by inland patches or depressions in the terrain with no direct opening to the surrounding ocean, or open, when directly connected to the nearby lagoon and often represented by small bays delimited by mangrove vegetation (Cerri et al. 2024). Open mangroves in particular are subject to daily tidal cycles that can transport nutrients and planktonic organisms (Shadiya et al. 2016) and contribute to the inland migration of various small fish and invertebrates. These organisms represent a valuable food source for higher predators, including eagle rays, which can exploit these delimited feeding grounds to ensure the presence of molluscs (Cerri et al. 2024; Louis, Bino, et al. 2024) on which they prey (Ajemian et al. 2012; Serrano‐Flores et al. 2019). Moreover, the limited water depth in these environments simultaneously restricts access to large predators of juvenile rays, such as adult pelagic sharks (Araujo et al. 2024), and provides shelter to their prey. Nevertheless, the role of mangroves for *A. ocellatus* and other batoids remains largely underestimated, partially due to the difficulty of conducting in‐water surveys in turbid and structurally complex habitats.

Knowledge gaps regarding migration patterns, aggregation sites, and habitat usage remain for all Aetobatidae species, despite eagle rays being widely distributed and charismatic organisms that attract attention during opportunistic observation, as demonstrated by the numerous sightings reported online (Araujo et al. 2020). Nevertheless, the generally elusive behaviour of *A. ocellatus* and similar species (Berthe et al. 2016), coupled with documented sensitivity to noise and anthropogenic disturbance (Berthe and Lecchini 2016), further constrains traditional survey approaches and limits the effectiveness of boat‐based or diver‐based monitoring in situ. Although the specimen in this study appeared largely undisturbed by the presence of the UAV when flown at ≥ 3 m AGL, the documented avoidance responses to boat noise (Berthe and Lecchini 2016), and in our case from human in water presence, suggest that future UAV surveys targeting batoids in similar environments should consider operating at minimum altitudes of ≥ 20 m where water clarity and image resolution permit, adopt slower lateral approach speeds, and limit repeated overflight of individual animals to minimise behavioural disruption. Despite that, UAV‐based observations represent a valuable, low‐impact tool for detecting elasmobranchs in shallow coastal environments, enabling high‐resolution coverage of areas otherwise difficult to access without bias linked to anthropogenic‐linked disturbances (Linchant et al. 2015). This is particularly relevant in remote regions such as the Maldives, where mangrove habitats are limited in extent, highly fragmented, and classified as threatened (Cerri et al. 2024), and where, therefore, in‐water efforts to collect relevant data often require intensification.

Moreover, UAVs are rapidly expanding in tourist videography, making drone‐generated videos among the most prevalent on social media (Vujičić et al. 2022). Open coastal landscapes, islands, and shallow embayments are the preferred environments (Chen et al. 2020), and because overflight and line transect are the most common techniques in UAV animal monitoring (Linchant et al. 2015), they can be easily applied by the general public with little to no experience in wildlife assessments. In fact, citizen science contributions have already proven effective for monitoring and assessing eagle rays. The extension of the cryptic *Aetomylaeus vespertilio* (Bleeker, 1852) has been documented in the Indo‐Pacific region through analysis of social media data, and additional records have been added from countries previously unreported (Araujo et al. 2020). Long‐term monitoring of citizen scientists identified an increase in the probability of sightings of numerous batoids, including *A. ocellatus* , in French Polynesia over the last decade (Séguigne et al. 2023). Photo‐identification of material submitted through a local citizen science programme has also been used to assess the spatial and temporal distribution of *A. narinari* (Euphrasen, 1790) in eastern Mexico (Cerutti‐Pereyra et al. 2018).

In addition to the ecological value of the observation, the study's strength is the confirmation that commercially available consumer‐grade UAVs, combined with citizen science contributions, can detect and document elusive elasmobranch species in challenging habitats during a single targeted observation. Some key limitations should be considered to guide the design of follow‐up studies, including the narrow temporal window of observation (~50 min across two flights), the measurement uncertainty inherent in UAV‐based methods without calibrated underwater reference targets and the absence of systematic tidal sampling across multiple visits. At this time, these factors preclude the assessment of fine‐scale movement and habitat use, but the future integration of repeated UAV surveys across tidal cycles, acoustic or satellite tagging, and dedicated in‐water connected analyses will provide complementary data on residency patterns, frequency of use, and seasonal variations.

Mangroves of the Maldives are critically endangered and under increasing threat from coastal development and pollution (Cerri et al. 2025), yet they remain scarcely studied from a biodiversity standpoint (Cerri et al. 2024; Louis, Cerri, and Galli 2024; Nicolai et al. 2025). These findings further reinforce the ecological importance of mangrove areas, especially in the Maldives, as potential nurseries and refuge habitats for juvenile marine species, including threatened elasmobranch species, such as the elusive *A. ocellatus*. Further research is needed to fully unravel the extent and specifics of animal–habitat relationships, but, in light of the limited spatial extent and high conservation concern for these ecosystems, documenting their use by juvenile elasmobranchs is crucial to promptly inform management and to develop effective conservation measures. Moreover, integrating citizen science into the ecological monitoring framework through accessible, repeatable technologies, such as UAVs, provides a large‐scale data supply that would otherwise be difficult for a limited scientific community to achieve. The use of publicly available photos and videos collected by the general public, albeit limited in the quantity of information they contain, can help fill the persistent knowledge gap and support evidence‐based conservation of both mangrove habitats and the species that depend on them.

## Author Contributions

**Giulia Senna:** conceptualization (equal), investigation (lead), writing – original draft (equal). **Jacopo Gobbato:** conceptualization (supporting), writing – review and editing (lead). **Yohan Didier Louis:** supervision (lead), writing – review and editing (lead). **Federico Cerri:** investigation (supporting), writing – review and editing (supporting). **Giorgia Gobbato:** investigation (supporting). **Rossella Nicolai:** investigation (supporting), writing – review and editing (supporting). **Jana Pažin:** investigation (supporting). **Andrea Parmegiani:** writing – review and editing (supporting). **Shazla Mohamed:** project administration (equal). **Paolo Galli:** funding acquisition (equal), supervision (equal).

## Funding

The authors have nothing to report.

## Conflicts of Interest

The authors declare no conflicts of interest.

## Supporting information


**Supporting Information: S1.** Smartphone‐recorded video of two *Aetobatus ocellatus* individuals swimming together in the mangrove embayment.

**Supporting Information: S2.** Underwater video of juvenile *Aetobatus ocellatus* with close‐up dorsal and side view.

## Data Availability

All further data is provided through Supporting Information.
